# Surface patches on recombinant erythropoietin predict protein solubility: engineering proteins to minimise aggregation

**DOI:** 10.1186/s12896-019-0520-z

**Published:** 2019-05-09

**Authors:** M. Alejandro Carballo-Amador, Edward A. McKenzie, Alan J. Dickson, Jim Warwicker

**Affiliations:** 1 Facultad de Ciencias, Universidad Autónoma de Baja California, Km. 103 Carretera Tijuana–Ensenada, Pedregal Playitas, 22860 Ensenada, Baja California Mexico; 2 School of Chemistry, Manchester Institute of Biotechnology, University of Manchester, 131 Princess Street, Manchester, M1 7DN UK; 3 Faculty of Science and Engineering, Manchester Institute of Biotechnology, University of Manchester, 131 Princess Street, Manchester, M1 7DN UK

**Keywords:** Protein solubility, Protein aggregates, Inclusion bodies, Erythropoietin, Solubility prediction, Protein expression

## Abstract

**Background:**

Protein solubility characteristics are important determinants of success for recombinant proteins in relation to expression, purification, storage and administration. *Escherichia coli* offers a cost-efficient expression system. An important limitation, whether for biophysical studies or industrial-scale production, is the formation of insoluble protein aggregates in the cytoplasm. Several strategies have been implemented to improve soluble expression, ranging from modification of culture conditions to inclusion of solubility-enhancing tags.

**Results:**

Surface patch analysis has been applied to predict amino acid changes that can alter the solubility of expressed recombinant human erythropoietin (rHuEPO) in *E. coli*, a factor that has importance for both yield and subsequent downstream processing of recombinant proteins. A set of rHuEPO proteins (rHuEPO E13K, F48D, R150D, and F48D/R150D) was designed (from the framework of wild-type protein, rHuEPO WT, via amino acid mutations) that varied in terms of positively-charged patches. A variant predicted to promote aggregation (rHuEPO E13K) decreased solubility significantly compared to rHuEPO WT. In contrast, variants predicted to diminish aggregation (rHuEPO F48D, R150D, and F48D/R150D) increased solubility up to 60% in relation to rHuEPO WT.

**Conclusions:**

These findings are discussed in the wider context of biophysical calculations applied to the family of EPO orthologues, yielding a diverse range of calculated values. It is suggested that combining such calculations with naturally-occurring sequence variation, and 3D model generation, could lead to a valuable tool for protein solubility design.

**Electronic supplementary material:**

The online version of this article (10.1186/s12896-019-0520-z) contains supplementary material, which is available to authorized users.

## Background

Biological systems have evolved by orchestration of molecular interactions, with proteins as key elements. In the circulatory system 2.4 million red blood cells are replaced every second in human adults [1]. This requires stable and efficient regulation to fulfil this demand. Erythropoietin (EPO), the main glycoprotein behind this task, regulates the growth and proliferation of red blood cell progenitors [2]. EPO is one of the top-selling therapeutics [3], providing therapy for millions of patients. There remains a continued demand to make EPO at large-scale and to increase the economic efficiency and, hence, availability to patients. Under this scheme, EPO has been a successful example of a biosimilar available in the market [3, 4]. Human erythropoietin (HuEPO) consists of 166 amino acid residues, in a structure that includes three N-linked glycosylation sites (N24, N38 and N83), an O-linked glycosylation (S126) and two disulphide bonds (C7-C161 and C29-C33) [5]. These complex post-translational modifications (PTMs) are the main challenge for expression of HuEPO in heterologous expression systems [6], and in the cost-efficient *E. coli* system none of these PTMs are effectively incorporated in the cytoplasmic environment. However, the activity of a non-glycosylated version of HuEPO expressed in *E. coli* has been proved in in vitro cell proliferation assays [7]. Using bacteria to produce recombinant proteins efficiently entails a significant challenge since cysteine mispairing may lead to misfolding and low yields [8]. In order to overcome this challenge, engineered strains of *E. coli* have been developed such as the SHuffle system [9]. In this *E. coli* strain the cytoplasmic environment is altered by the overexpression of DsbC disulphide bond isomerase, and by deletion of two reductases (glutaredoxin [gor] and thioredoxin [trxB]).

Glycosylation of HuEPO contributes ~ 40% of the overall molecular mass, improving stability and solubility of the molecule as a therapeutic [10–12]. Lack of glycosylation in recombinant HuEPO (rHuEPO) derived from *E. coli* leads to aggregation during expression and, potentially, during subsequent purification, storage and delivery [13]. This protein aggregation phenomenon during expression in *E. coli* leads to incorporation into inclusion bodies (IBs), from the interactions of partially folded, misfolded or unfolded recombinant proteins in the cytoplasm [8]. Surface charge engineering has been illustrated by mutation of the three N-glycosylation sites on HuEPO to lysine (N24K, N38K and N83K), increasing net charge. This engineering decreased IB formation and facilitated the purification of protein to provide the rHuEPO crystal structure [13, 14].

Protein aggregation can involve chemical aggregation, such as disulphide bond formation, and/or physical aggregation, such as non-covalent interactions between hydrophobic surfaces [15]. Several approaches have been undertaken to diminish hydrophobic patches on the surface to prevent protein aggregation [16–23]. In this context, improved expression, stability and solubility of rHuEPO and granulocyte colony-stimulating factor (G-CSF) has been generated by application of the in vitro ribosome display technique, in combination with three parallel selection pressures (reducing agent, elevated temperature and hydrophobic interaction chromatography matrices) [24]. In the case of rHuEPO, a variant encoding four mutations resulted in a form that was less prone to aggregation [24]. Furthermore, the application of fusion tags to improve rHuEPO solubility has been successful [7, 25]. Some of these fusion partners, including NusA and maltose-binding protein (MBP), have large negatively-charged areas that may be involved in promotion of folding of the target protein by limiting protein aggregation [26]. Engineering of negatively-charged areas on protein surfaces is gaining strength as an approach for increasing solubility [27–31].

Here we report a novel experimental approach targeted at improvement of rHuEPO solubility for expression in *E. coli*, following the observation that soluble expression of proteins is inversely correlated with the size of the largest positively-charged patch on the protein surface [29, 32]. This result is based on data from cell-free expression [33], and here we test the hypothesis by mapping surface charge of rHuEPO, focusing on modulation of positively-charged patches through mutagenesis. A set of mutants has been generated, ranging from more (rHuEPO E13K) to less positively-charged surface patches (rHuEPO F48D, R150D and F48D/R150D), compared to natural (wild type) rHuEPO (rHuEPO WT). Experimental results support the prediction, i.e. largest positively-charged patch size correlated with the degree of protein aggregation in the cytoplasm of *E. coli*. Further application of this approach, particularly in the context of natural protein surface variation, could improve the rational design of proteins with enhanced solubility in cytoplasmic expression.

## Results

### Redesign of rHuEPO WT for altered charge surface

**Fig. 1:**
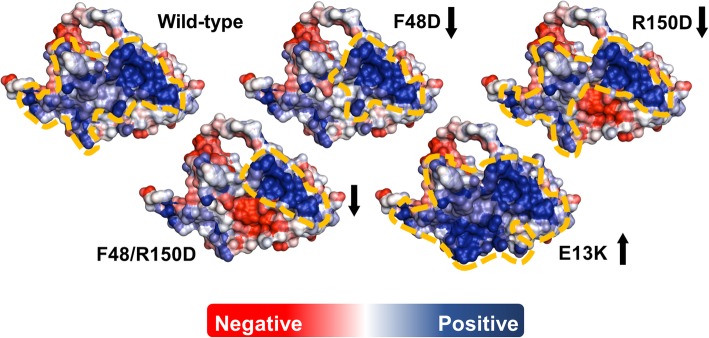
HuEPO wild-type and variants surface illustration showing the electrostatic potential patches [29]. Amino acids in positive patches are represented by blue, non-charged patches by white and negatively charged by red colour, respectively, with dashed yellow contours drawn in to delineate the largest positive patches

A published algorithm [29] was used to identify amino acids of rHuEPO for which mutation could be predicted to alter solubility (Table 1). The method is based on an observed correlation between positive charge patches and insolubility [29] for data derived in a cell-free expression system [33]. Design for improved solubility, therefore, involved identification and reduction of the larger positively-charged patch. For the protein variants shown in Table 1, the substitution R150D gave a lowered positive patch size and was predicted to be more soluble than rHuEPO WT. In contrast, substitution E13K had an increased positive patch compared with wild type and was predicted to generate a less soluble product. Both of these sites lie on the protein surface. A third site was chosen to introduce a negative charge (F48D), rather than make a charge swap, and decrease the size of the largest positive patch. It was recognized that although F48 was also on the surface, this mutation might present a more challenging mutation structurally since the phenylalanine ring covers in part the hydrophobic sidechains of V46 and L155. Figure 1 shows the single site mutations and one double site mutation employed in this study, and their charge surfaces in comparison with wild type rHuEPO. Amino acid conservation analysis [34–36] showed that R150 is relatively conserved across evolution, but E13 and F48 showed less conservation (see Additional file 1: Figure S1). Previous site-directed mutagenesis of these residues had shown no alteration of the folded state of rHuEPO structure [37].

**Table 1:** Predicted solubilities of recombinant human erythropoietin. Solubility profile was defined as described in Chan et al. [29]. Positive patch sizes are divided by that best separating soluble and insoluble datasets [33], above 1.0 implies predicted insolubility

Protein	Pos patch ratio to threshold	Prediction
rHuEPO wild-type	1.49	Insoluble
rHuEPO F48D	0.75	Soluble
rHuEPO R150D	0.61	Soluble
rHuEPO F48D/R150D	0.47	Soluble
rHuEPO E13K	2.47	Insoluble

### Soluble expression of rHuEPO is modulated in line with the predictions

**Fig. 2:**
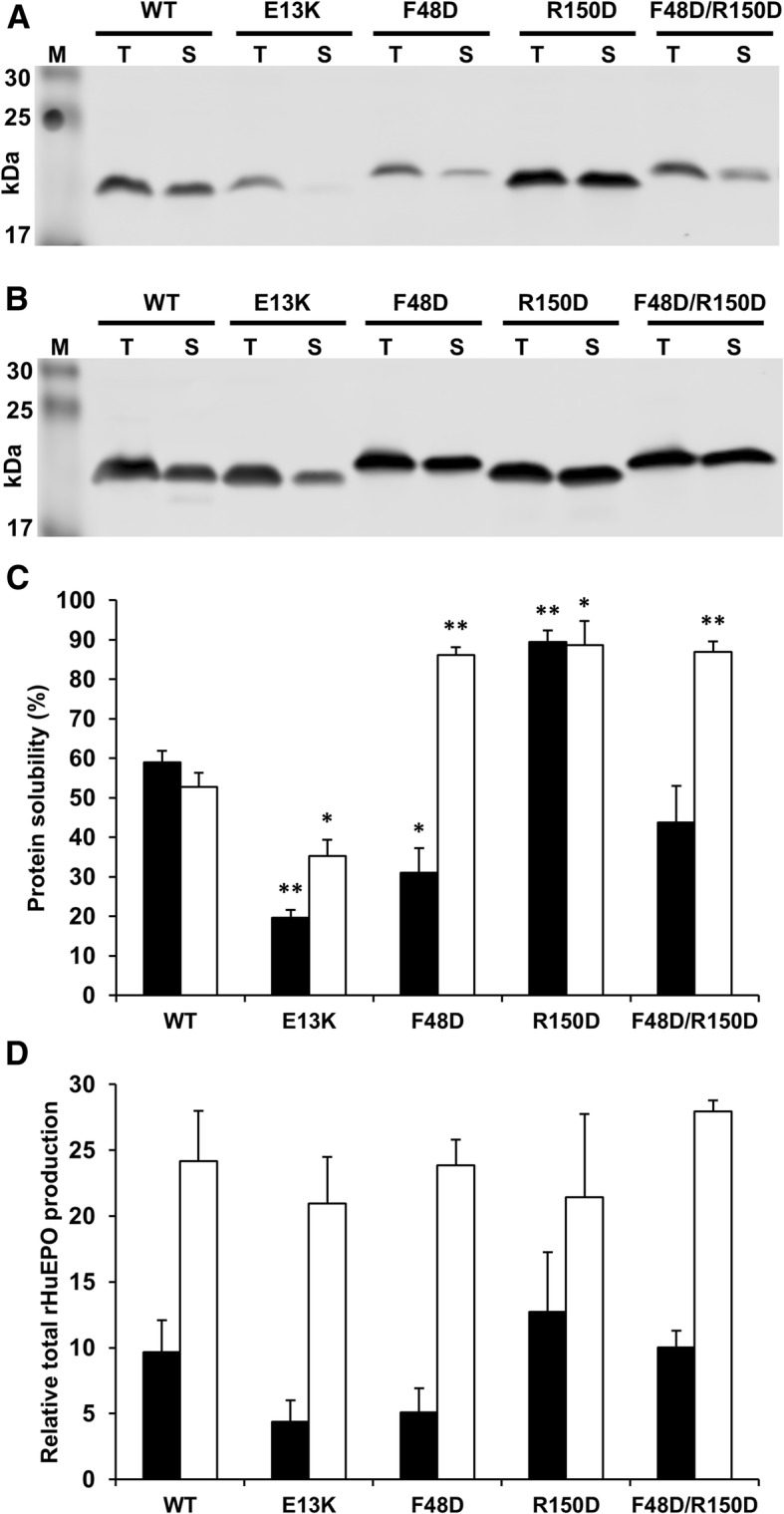
Western blot of rHuEPO expression and solubility. (**a**-**b**) Equal volumes of total protein and soluble fraction were probed with Mouse anti-polyHis antibody and imaged with the Odyssey Imaging System for BL21 (DE3) pLysS (**a**) and SHuffle (**b**) strains. (**c**) Experimental solubility was determined by the distribution of rHuEPO between soluble and inclusion body fraction in *E. coli*. (**d**) Relative total rHuEPO production (arbitrary units). Error bars represent the + SEM for measurements in triplicate; statistical significance was calculated using a two-sided unpaired t-test (*P < 0.05, ** *P* < 0.01). BL21 (DE3) pLysS (■); SHuffle (□)

The expression and solubility of rHuEPO variants were studied under low induction conditions (Fig. 2). The total expression of rHuEPO was approximately equivalent across WT and mutant constructs, particularly in the SHuffle system (Fig. 2d). Protein solubility, assessed as the ratio of EPO detected in soluble and total EPO fraction, agrees with the prediction for all 4 mutant constructs in the SHuffle system, but for only 2 of the 4 variants in the BL21 system, the exceptions being those involving mutation at F48 i.e. rHuEPO F48D and F48D/R150D (Fig. 2c).

**Fig. 3:**
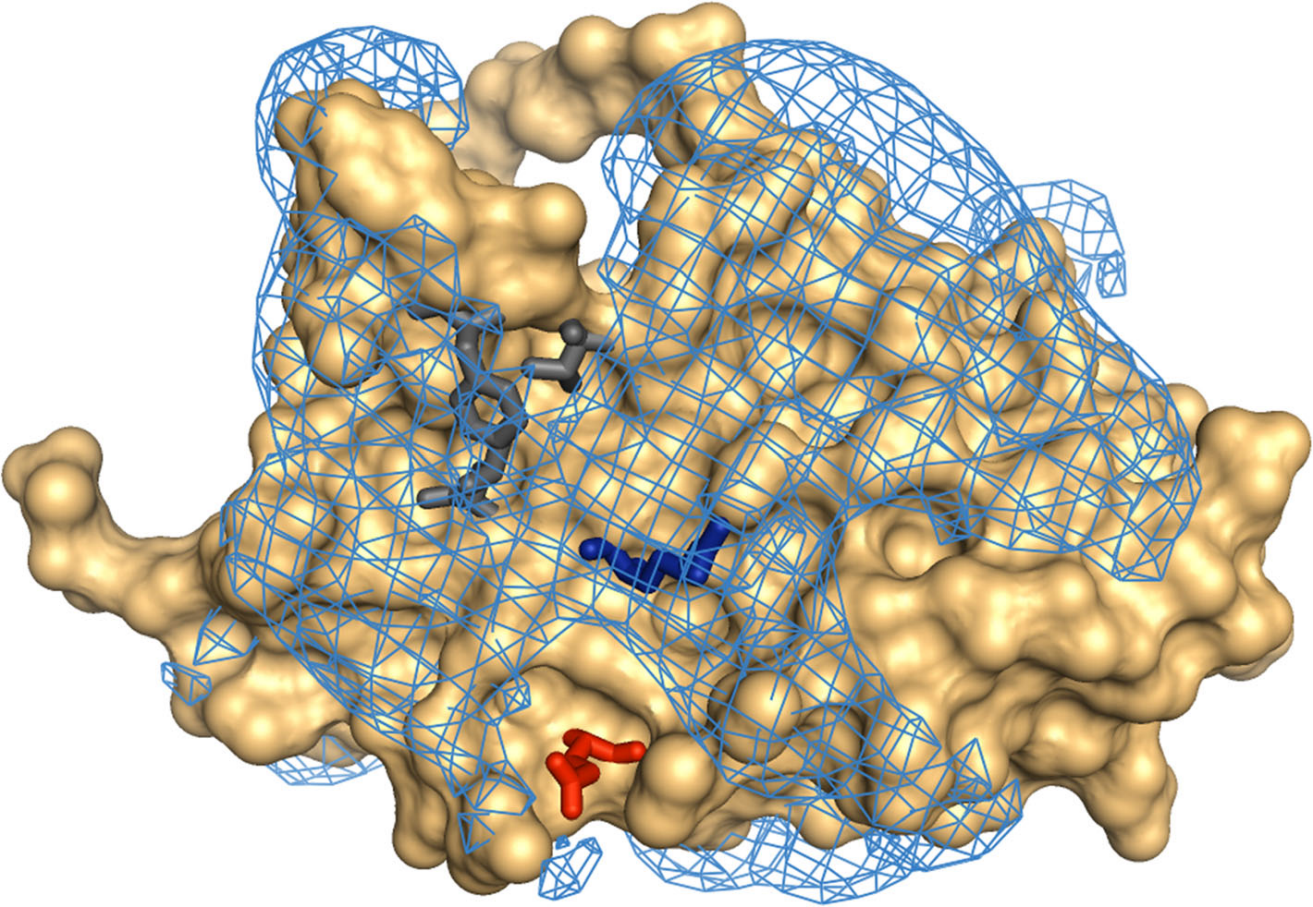
Mutated residues on the surface of HuEPO. Molecular surface is shown (orange), with positive electrostatic field contoured at 30 mV (blue mesh). Residues E13 (red), F48 (grey) and R150 (blue) are shown in sticks, rather than surface representation. Also drawn are the non-polar residues V46 and L155 that are covered by F48 in the WT structure

That the two-way charge swap tests (R150D and E13K) match predictions in both expression systems used is encouraging in terms of applying a correlation [29] learned from a large dataset, albeit in cell-free expression [33]. With regard to the general mechanism through which charge may play a role in determination of solubility, there is an increasing body of work that indicates that negatively-charged residues (rather that positively-charged residues) are more favourable for protein solubility [27, 30, 38–41]. Whatever the molecular mechanism by which the engineering of charged patches alters solubility, it may be associated with attainment of the native, or a near native state. This would be consistent with the observation here that charge-based predictions are matched for SHuffle but not with the BL21 system. Expression in the SHuffle system will, in relative terms, favour correct formation of the two disulphide bonds in the folded recombinant protein. The F48D mutation, apart from introducing negative charge, may expose more non-polar surface than it removes, due to the sidechains of V46 and L155 that lie beneath F48 (Fig. 3). It would be expected that the BL21 strain should be more susceptible than the SHuffle strain to this exposure, since it would be less able to refold partially unfolded protein. This difference may underpin the solubility data for F48D and F48D/R150D (Fig. 2c). Although the aspartic acid sidechain introduced in the F48D mutant could in principle alter pH-dependent properties, protein expression and measurements are made at neutral pH, for which a negative charge will be carried by the F48D carboxylate group.

### Bioinformatics and solubility engineering

**Fig. 4:**
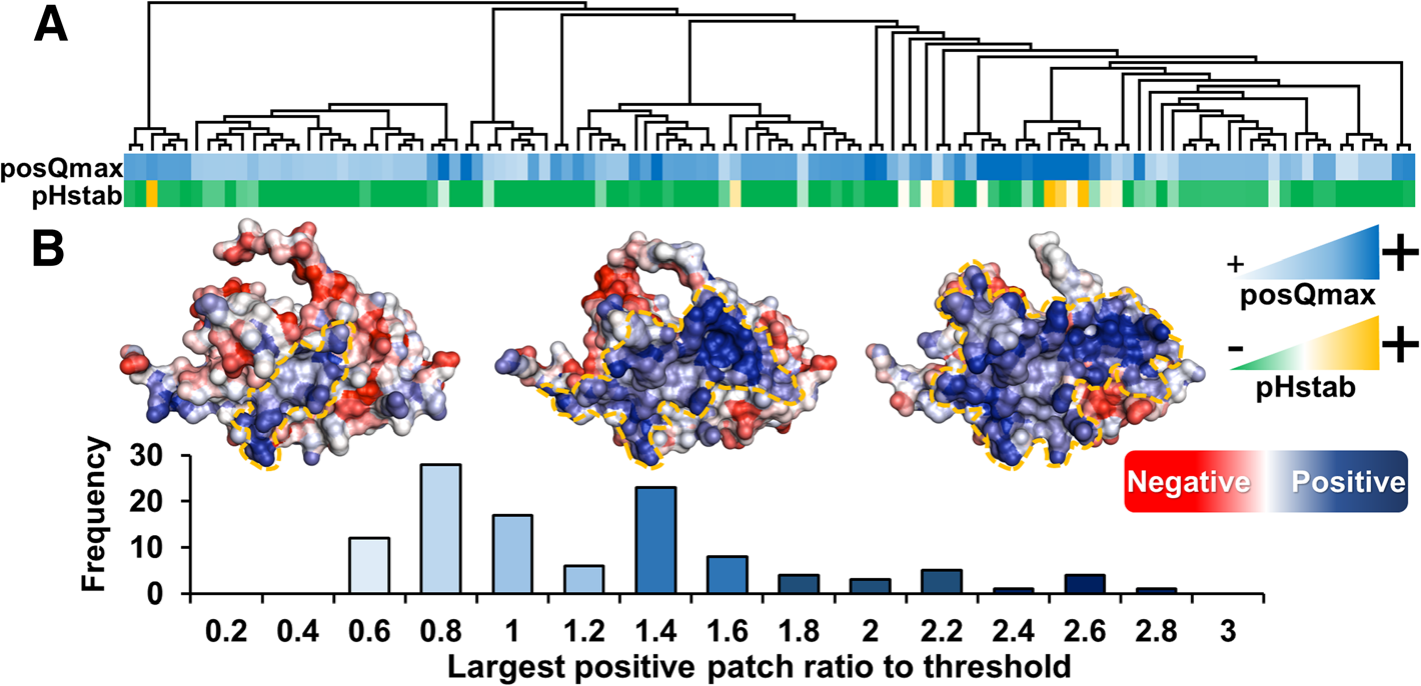
Positively-charged patches and predicted stability in EPO orthologues. (**a**) A sequence-based phylogenetic tree is combined with positive patch and stability calculations. Colour coding for posQmax varies from lighter to darker blue as the calculated largest positive patch increases for an EPO orthologue (same colour code for histogram in panel **b**). Predicted pH-dependent contribution to folded state stability (pHstab) varies from yellow (positive, unfavourable) to green (negative, favourable). (**b**) Distribution of largest positive patch ratios to the threshold for the 115 EPO orthologues, showing that surface charge changes substantially. HuEPO is located roughly in the centre of the distribution. Frequency is the number of EPO orthologues having a largest positive patch ratio to threshold, in the given x-axis bin

Having found that charge surface properties, and charge contributions to folded state stability, are factors that commonly arise in experimental studies aimed at improvement of EPO production, we assessed how these properties varied between EPO orthologues. The largest positively-charged patches, and the predicted contribution of ionisable group charge interactions to folded state stability at pH 7, were calculated for 115 EPO homologues found through a BLAST [42] search, and passed through a comparative modelling pipeline. Positive patches are distributed over a surprisingly large range (viewed as a distribution or as individual examples, Fig. 4). Since it is not an evolutionarily conserved property, positive patch size presumably only becomes relevant for protein expression at the higher levels of over-expression, in comparison with expression in nature. As a general consideration, for over-expression, the range of values permissible naturally could present an important design feature for protein production (especially in relation to development of novel format species). A heat map, clustered according to pairwise sequence identity in Clustal [43], shows that positive patch sizes tend to cluster together, with occasional larger transitions (Fig. 4a). This indicates that a small number of mutations, or even a single mutation as in the current study, can significantly alter the charge distribution of a protein.

## Discussion

When expressed in *E. coli* rHuEPO WT tended to form insoluble protein aggregates in inclusion bodies [14]. The use of sub-optimal temperature and lower inducer concentrations has been argued to be a more appropriate approach to assess protein folding (and hence solubility) of recombinant proteins in *E. coli* [44]. Low induction conditions (decreased temperature, lower IPTG challenge) decreased cell growth and the elongation rate of translation [45] and induced chaperone activity and protein folding capability [46]. These responses led to better folding and less degradation [47] and offered a more refined system to investigate the consequences of variant structures on the expression and solubility of recombinant protein expression in *E. coli*.

Our understanding of IB formation and subsequent recovery of native protein is increasing [48, 49]. Important factors include the effects of a particular protein on metabolic burden in *E. coli* [50], chaperone and codon optimisation [51, 52], particular genetic loci [53], and solubility tag and medium engineering of the expression system [54]. However, there are still unknowns in what dictates IB formation for particular proteins. With regard to solubility in cell-free expression [33], a model was put forward [29] as one potential explanation of the correlation, involving charge interactions between the positive charge surface of a protein and large concentrations of a biological macro-anion i.e. mRNA. The current work does not address the underlying model. Other studies have highlighted the importance of charge properties in the production of EPO, from the incorporation of three lysines to remove the N-linked glycosylation sites and modify pI [14], to recent work in which the contribution of positively-charged patches was highlighted [55]. In the latter case, it was reasoned that improved solubility properties associated with increased ionic strength could be a result of lessening of repulsion within clusters of basic sidechains. The positively-charged region is the same as that studied here. However, the rationale for modification of aggregation properties is different, i.e. native state stabilization [55] compared with reduction of self-association (current work). The systems are quite different, purified protein [55] compared with cytoplasmic expression in *E. coli* (current work), and the suggested molecular mechanisms in each case remain unproven at this stage. It is intriguing that the same positively-charged region on EPO has featured in a predictive study (current work) and in a post-hoc rationalization [55]. Further, this patch has also been implicated in a selection mutagenesis screen for EPO variants with improved stability [24]. Ribosome display led to a variant that incorporated mutations at 4 sites, and exhibited a decreased aggregation in accelerated shelf-life studies. One of the mutations in that variant was G158E, introducing a negative charge into the largest positively-charged patch [24].

The ionic strength dependence study of Banks (2015) implied that charge-charge interactions are delicately balanced in EPO (at pH 6.9), and thus predicted ionisable group contributions to folded state stability are also shown in the heat map format (at pH 7.0). It is relatively rare for proteins to have a predicted contribution (or indeed a measured contribution) that is net unfavourable as shown for a few examples here (Fig. 4a). This is likely to be correlated with the observation that increasing ionic strength improves the folded state stability for EPO, since unfavourable charge-charge interactions will be diminished [55]. Again, a relatively simple bioinformatics calculation shows molecular detail that is likely to underpin observed changes in EPO production, and furthermore it can guide design for improved production.

Importantly, no account has been taken of glycosylation in these interpretations, since it is difficult to model the conformations of these components with respect to the protein. The net negative charge carried by the glycosyl groups is emphasized by the change in pI of EPO from 9.2 to 4.4 upon glycosylation [10]. Interactions between sialic acid groups and positive charges on the protein are likely to improve the stabilizing component of the charge-charge balance. It should be emphasized that when charge-charge interactions contribute relatively little in net terms at neutral pH, this is generally the result of favourable and unfavourable terms cancelling, not due to a complete absence of ionisable group interactions. Such a situation lends itself to modification by relatively small changes in amino acid sequence (depending on location of those changes in the structure). In the heat map for EPO homologue stability contributions from charge-charge interactions (Fig. 4a), similar contributions generally cluster together (within the sequence-based phylogenetic tree), but there are also some abrupt changes, emphasizing the sequence-sensitivity. In general terms, the least stable contributions tend to group with the most positive patches (and vice versa). This may support the rationalization given for improved resistance to aggregation as ionic strength is increased for pure EPO [55], in that larger positively-charged patches are predicted to associate with less stable folded proteins.

## Conclusions

Solubility prediction for proteins has been the focus of several groups in the last 25 years [38–41, 56–59]. Here, a method developed in our group [29] has been tested. Mutations of rHuEPO gave experimental results in line with predictions, excepting F48D in one of the two expression systems used. The F48D mutation stands apart from the other (charge swap) variants, in that it is likely to alter the hydrophobicity. It is therefore plausible that F48D leads to reduced folding efficiency, a suggestion consistent with an improved solubility in the SHuffle system, compared with reduced solubility in the BL21 strain. Further purification might allow a better structural understanding of these mutations, however, native purification has not been possible [7, 11, 13, 14]. Finding that the region of EPO targeted here also appears in selection mutagenesis for improved stability [24] and a recent study of ionic strength effects [55], bioinformatics was employed to reveal the extent of variation in EPO homologues. Interestingly, both the largest positively-charged patch and the predicted contribution of ionisable group interactions to stability (pH 7) vary substantially through evolution, suggesting that the solubility of these EPO homologues, if over-expressed, would be divergent. Indeed, even small changes in amino acid sequence can lead to relatively large changes in solubility. Whilst it may not always be feasible to engineer a human protein across species (e.g. considering immunogenicity of a therapeutic protein), biophysical calculations for a set of homologues could guide design of protein with enhanced stability and solubility for large-scale expression, if incorporated at a sufficiently early point in the design cycle.

## Methods

### rHuEPO solubility profile and mutant design

The PyMOL Molecular Graphics System version 1.3 [60] and Swiss-PdbViewer 4.0.1 [61] were used to analyse rHuEPO structural and sequence features. Protein solubility predictions were calculated using an algorithm developed in our group [29] (see Additional file 2: Table S1 and Additional file 3: Table S2). The algorithm computes structured-based parameters, including the sizes of positively- and negatively-charged patches, when the electrostatic potential field is contoured at + 25 mV or − 25 mV. It also gives the size of the largest patch for which all points are between − 25 mV and + 25 mV (effectively non-charged). The principal prediction [29] is generated from the ratio of the largest positively-charged patch to a threshold, but a supplemental prediction from the combination of positive and non-charged patches is also made. Thresholds were calculated from a dataset of experimental solubilities determined for cell-free expression of *E. coli* proteins [33], as the value of a parameter that best separates less and more soluble proteins. Proteins with larger positive patches are predicted as less soluble, and have a ratio to threshold above 1.0. Where the ratio to threshold is below 1.0, a protein is predicted as soluble. Current work concentrates on positive patches (posQ), since this structure-based feature gives the best separation [29]. Substitutions to modify posQ, based on the protein data bank [62] file 1EER [63] were carried out in Swiss-PdbViewer, and the resulting structures analysed for modification of the largest positive patch (Table 1). This in silico mutational screening gave the following candidate mutations: rHuEPO E13K, rHuEPO F48D, rHuEPO R150D and the double mutant rHuEPO F48D/R150D. Aspartic acid was selected for the introduction of negative charge, in preference to glutamic acid, due to its shorter side chain, lowering the possibility of non-specific interactions with surrounding side chains. Calculations were performed using a modified crystal structure of an analogue rHuEPO taken from the 1EER PDB entry in order to maintain consistency with our experimental and native rHuEPO cDNA (K24 N, K38 N, K83 N, N121P and S122P).

### Bioinformatics analysis of rHuEPO surface and stability

Multiple sequence alignments and surface mapping coloured by residue conservation were performed using ConSurf with default parameters [34–36], using the UniRef90 database [64], which removes redundancy at 90% sequence identity (see Additional file 1: Figure S1). A separate structural study of EPO homologues was made with 115 models generated from a Clustal alignment [43] using a sidechain replacement method [65] for comparative modelling. Input to the Clustal alignment was from a search for EPO orthologues with BLAST [42], followed by manual checking of EPO annotation. Patch calculations were made based on the comparative models, to give a view of EPO variation over species.

The set of EPO comparative models was also used to estimate the contribution of ionisable groups (i.e. the pH-dependent stability term) to folded state stability at pH 7. A Debye-Hückel model for interactions between ionisable group charges, at 0.15 M ionic strength was used, with Monte Carlo sampling of protonation states to derive the pH-dependent term. This modelling follows previous methodology [66], and includes subtraction of an estimated unfolded state set of interactions to arrive at the predicted pH-dependent term for folded state compared with unfolded state.

### Construction of rHuEPO mutants and expression vectors

Human erythropoietin cDNA was amplified from a pre-existing mammalian expression vector by applying primers containing the restriction sites 5′-*BamHI* and 3′-*EcoRI*. The PCR fragment (lacking signal peptide) was subcloned into a pHis vector. This plasmid is a modified version of the commercial pET-16b vector (Novagen). The gene sequence for each plasmid was as follows: 5′-6xHis-Thrombin cleavage site-*BamHI*-rHuEPO-*EcoRI*-3′. rHuEPO mutations were introduced using the GENEART Site-Directed Mutagenesis System with the enzyme AccuPrime *Pfx* (Invitrogen).

### Protein expression and solubility assay

The bacterial cell lines used in this study were *Escherichia coli* BL21 (DE3) pLysS and SHuffle (New England BioLabs). Bacterial strains were transformed with the pHis-rHuEPO plasmids. Transformed cells were grown overnight in 5 ml working volume of Luria-Bertani (LB) medium (10 g tryptone, 5 g yeast extract, 5 g NaCl) containing 100 μg/ml ampicillin at 37 °C with shaking at 220 rpm. In addition, BL21 (DE3) pLysS were grown in the presence of chloramphenicol (50 μg/ml) in order to preserve the pLysS plasmid. On the following day, 1 ml of pre-culture was transferred to 50 ml 2% (v/v) LB supplemented with 2% (w/v) glucose with 100 μg/ml ampicillin in 250 ml shake flasks. Experiments were performed in triplicate. Shake flasks were incubated at a constant temperature of 25 °C, with shaking at 180 rpm. Bacteria were grown to an OD_600_ of approximately 0.6–0.8. Protein expression was induced by the addition of IPTG (0.05 mM, final). After 5 h, cultures were centrifuged at 6500 g for 15 min at 4 °C. Bacterial pellets were suspended in 5 ml of lysis buffer (25 mM Tris pH 7.5, 150 mM NaCl, 1% [v/v] Triton X-100) and were stored at − 20 °C until future use. The cells were disrupted by six sonication cycles of 30 s at 20% amplitude and then allowed to cool for 30 s on ice water bath. Separation of soluble and total fractions was performed by centrifugation at 18,000 g for 30 min at 4 °C of 1 ml of each sample from the whole cell lysate. The supernatants were collected and handled as the soluble fraction. An additional 1 ml of each lysate sample was processed as the total fraction, and rHuEPO solubility was calculated by densitometric ratio of protein detected in soluble and total fractions.

Proteins in soluble and total fractions were separated by sodium dodecyl sulphate-polyacrylamide gel electrophoresis (SDS-PAGE) with 12% (w/v) acrylamide using the Mini-PROTEAN Tetra Cell (BioRad). Samples containing equal volumes (20 μl) of protein were subjected to heat at 95 °C for 5 min in 6x denaturing buffer (375 mM Tris pH 6.8, 12% [w/v] SDS, 60% [v/v] glycerol, 0.06% [w/v] bromophenol blue, 5.5% [v/v] β-mercaptoethanol). Separated proteins were transferred to nitrocellulose membranes using a transblot semi-dry transfer cell (Bio-Rad) at 15 V for 45 min. Membranes were blocked overnight for non-specific binding in blocking buffer (5% [w/v] skimmed milk in TBS-Tween pH 7.4) at 4 °C with shaking. For detection of rHuEPO, a mouse anti-polyHis antibody (Sigma) was diluted 1:5000 in blocking buffer solution and the membrane was incubated in this solution for 2 h at room temperature with agitation. After three washes in TBS-Tween (5 min each time), samples were incubated with an IR-labelled secondary Donkey anti-Mouse IgG antibody (LI-COR) diluted 1:15000 in blocking buffer solution at room temperature for 45 min. Following incubation, the secondary antibody was removed and the membrane was washed three times. For IR detection, blots were imaged with the Odyssey Imaging System. Bands were quantified in Image Studio Lite software (LI-COR).

## Additional files

Additional file 1: **Figure S1**. Multiple alignment of HuEPO. (A) A surface map is coloured by residue conservation scores [34–36]. The image was rendered using PyMOL [60]. (B) Panel shows the same color-coding for conservation show in panel (A), but here applied to the amino acid sequence of rHuEPO. (PDF 1525 kb)

Additional file 2: **Table S1**. Positively-charged patches size profile of rHuEPO WT from the charged patch calculator. Complete screening of posQ ratio scores for the modified rHuEPO WT (PDB: 1EER) is shown. The largest positive patches are represented by blue (ratio > 1.0). Those proteins with ratio above 1.0 are predicted as insoluble and below 1.0 as soluble. The three targeted residues in this study are highlighted in red. Ratio: largest positively-charged patch (posQ) value from the charged patch calculator [29]. Charge patches: HYD, hydrophobic (non-charged); NEG, negatively-charged; POS, positively-charged. (PDF 478 kb)

Additional file 3: **Table S2**. Summary of the solubility screening of rHuEPO. Left column shows the complete mutational screening of all positive charge amino acids (i.e. arginine and lysine) within the largest positively-charged patch (posQ) for aspartic acid (D). Next two columns summarize a set of substitutions of any amino acid in the posQ for D. The column on the right shows all the negative charge residues (i.e. aspartic and glutamic acid) within the posQ for arginine or lysine. Those proteins with posQ ratio above 1.0 are predicted as insoluble and below 1.0 as soluble. Selected proteins for further site-directed mutagenesis are highlighted in red. (PDF 168 kb)

## Abbreviations

EPO: Erythropoietin
HuEPO: Human erythropoietin
IBs: Inclusion bodies
posQ: positive patches
PTMs: Post-translational modifications
rHuEPO: Recombinant human erythropoietin
WT: Wild-type protein
