# Supplementary material for: Surface patches on recombinant erythropoietin predict protein solubility: engineering proteins to minimise aggregation
Source: BMC Biotechnol. 2019 May 9;19:26. doi: 10.1186/s12896-019-0520-z (PMC6507049; doi:10.1186/s12896-019-0520-z)
Supplement: Supplementary file 1 — Figure S1. Multiple alignment of HuEPO. (A) A surface map is coloured by residue conservation scores [34–36]. The image was rendered using PyMOL [60]. (B) Panel shows the same color-coding for conservation show in panel (A), but here applied to the amino acid sequence of rHuEPO. (PDF 1525 kb) [file 12896_2019_520_MOESM1_ESM.pdf]

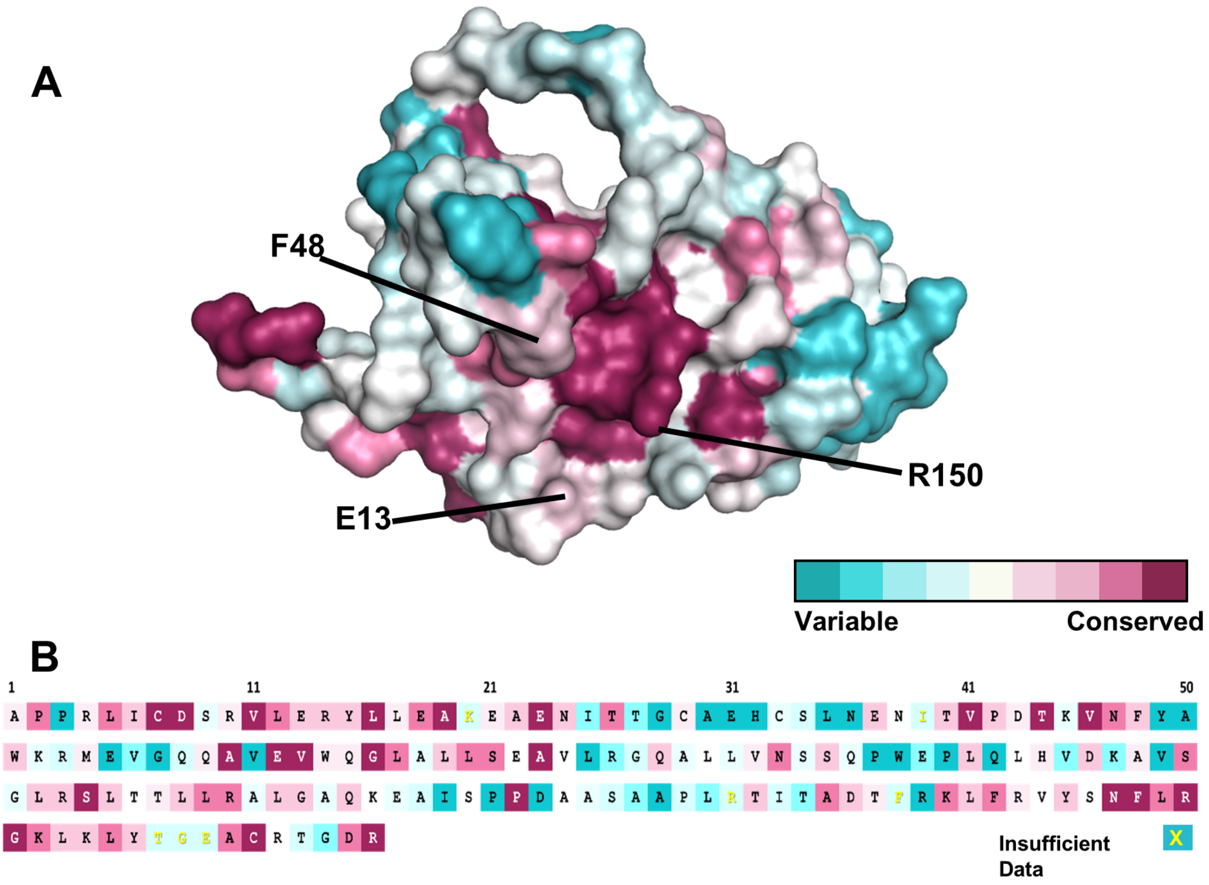

Additional file 1: Figure S1. Multiple alignment of HuEPO. (A) A surface map is coloured by residue conservation scores [34-36]. The image was rendered using PyMOL [60]. (B) Panel shows the same color-coding for conservation show in panel (A), but here applied to the amino acid sequence of rHuEPO.
