# Supplementary material for: Surface patches on recombinant erythropoietin predict protein solubility: engineering proteins to minimise aggregation
Source: BMC Biotechnol. 2019 May 9;19:26. doi: 10.1186/s12896-019-0520-z (PMC6507049; doi:10.1186/s12896-019-0520-z)
Supplement: Supplementary file 2 — Table S1. Positively-charged patches size profile of rHuEPO WT from the charged patch calculator. Complete screening of posQ ratio scores for the modified rHuEPO WT (PDB: 1EER) is shown. The largest positive patches are represented by blue (ratio > 1.0). Those proteins with ratio above 1.0 are predicted as insoluble and below 1.0 as soluble. The three targeted residues in this study are highlighted in red. Ratio: largest positively-charged patch (posQ) value from the charged patch calculator [29]. Charge patches: HYD, hydrophobic (non-charged); NEG, negatively-charged; POS, positively-charged. (PDF 478 kb) [file 12896_2019_520_MOESM2_ESM.pdf]

Additional file 2: Table S1. Positively-charged patches size profile of rHuEPO WT from the charged patch calculator. Complete screening of posQ ratio scores for the modified rHuEPO WT (PDB: 1EER) is shown. The largest positive patches are represented by blue (ratio > 1.0). Those proteins with ratio above 1.0 are predicted as insoluble and below 1.0 as soluble. The three targeted residues in this study are highlighted in red. Ratio: largest positively-charged patch (posQ) value from the charged patch calculator [29]. Charge patches: HYD, hydrophobic (non-charged); NEG, negatively-charged; POS, positively-charged.

Residue			Ratio																
ALA	1	POS	1.492	LEU	35	NEG		LEU	69	NEG		ARG	103	POS	0.477	THR	137	POS	1.492
PRO	2	POS	1.492	ASN	36	HYD		LEU	70	NEG		SER	104	HYD		PHE	138	NEG	
PRO	3	HYD		GLU	37	HYD		SER	71	HYD		LEU	105	POS	0.477	ARG	139	POS	1.492
ARG	4	POS	1.492	ASN	38	HYD		GLU	72	HYD		THR	106	POS	0.477	LYS	140	POS	1.492
LEU	5	POS	1.492	ILE	39	NEG		ALA	73	NEG		THR	107	POS	0.477	LEU	141	POS	1.492
ILE	6	POS	0.003	THR	40	NEG		VAL	74	HYD		LEU	108	HYD		PHE	142	POS	1.492
CYS	7	POS	1.492	VAL	41	HYD		LEU	75	HYD		LEU	109	HYD		ARG	143	HYD	
ASP	8	HYD		PRO	42	POS	0.022	ARG	76	HYD		ARG	110	POS	0.014	VAL	144	POS	1.492
SER	9	POS	1.492	ASP	43	HYD		GLY	77	NEG		ALA	111	HYD		TYR	145	HYD	
ARG	10	HYD		THR	44	POS	1.492	GLN	78	NEG		LEU	112	NEG		SER	146	HYD	
VAL	11	HYD		LYS	45	POS	1.492	ALA	79	HYD		GLY	113	NEG		ASN	147	POS	1.492
LEU	12	HYD		VAL	46	HYD		LEU	80	NEG		ALA	114	NEG		PHE	148	POS	1.492
GLU	13	POS	1.492	ASN	47	POS	1.492	LEU	81	NEG		GLN	115	POS	0.014	LEU	149	HYD	
ARG	14	HYD		PHE	48	POS	1.492	VAL	82	NEG		LYS	116	HYD		ARG	150	POS	1.492
TYR	15	NEG		TYR	49	POS	1.492	ASN	83	HYD		GLU	117	HYD		GLY	151	POS	1.492
LEU	16	HYD		ALA	50	HYD		SER	84	HYD		ALA	118	NEG		LYS	152	POS	1.492
LEU	17	NEG		TRP	51	HYD		SER	85	NEG		ILE	119	NEG		LEU	153	HYD	
GLU	18	HYD		LYS	52	NEG		GLN	86	NEG		SER	120	NEG		LYS	154	POS	1.492
ALA	19	POS	0.477	ARG	53	HYD		PRO	87	HYD		PRO	121	NEG		LEU	155	HYD	
LYS	20	POS	1.492	MET	54	HYD		TRP	88	POS	1.492	PRO	122	NEG		TYR	156	HYD	
GLU	21	NEG		GLU	55	HYD		GLU	89	NEG		ASP	123	NEG		THR	157	POS	1.492
ALA	22	POS	0.477	VAL	56	NEG		PRO	90	HYD		ALA	124	HYD		GLY	158	POS	1.492
GLU	23	POS	1.492	GLY	57	NEG		LEU	91	HYD		ALA	125	NEG		GLU	159	POS	1.492
ASN	24	HYD		GLN	58	NEG		GLN	92	HYD		SER	126	HYD		ALA	160	NEG	
ILE	25	HYD		GLN	59	NEG		LEU	93	HYD		ALA	127	HYD		CYS	161	POS	1.492
THR	26	HYD		ALA	60	NEG		HIS	94	POS	0.477	ALA	128	HYD		ARG	162	POS	1.492
THR	27	POS	1.492	VAL	61	HYD		VAL	95	POS	0.477	PRO	129	HYD		THR	163	POS	1.492
GLY	28	HYD		GLU	62	HYD		ASP	96	NEG		LEU	130	POS	0.025	GLY	164	POS	1.492
CYS	29	POS	1.492	VAL	63	HYD		LYS	97	HYD		ARG	131	HYD		ASP	165	NEG	
ALA	30	POS	1.492	TRP	64	HYD		ALA	98	POS	0.477	THR	132	POS	0.022	ARG	166	NEG	
GLU	31	HYD		GLN	65	HYD		VAL	99	HYD		ILE	133	POS	1.492				
HIS	32	NEG		GLY	66	HYD		SER	100	HYD		THR	134	POS	1.492				
CYS	33	NEG		LEU	67	HYD		GLY	101	NEG		ALA	135	POS	1.492				
SER	34	POS	1.492	ALA	68	HYD		LEU	102	HYD		ASP	136	POS	1.492				
