# Supplementary material for: Surface patches on recombinant erythropoietin predict protein solubility: engineering proteins to minimise aggregation
Source: BMC Biotechnol. 2019 May 9;19:26. doi: 10.1186/s12896-019-0520-z (PMC6507049; doi:10.1186/s12896-019-0520-z)
Supplement: Supplementary file 3 — Table S2. Summary of the solubility screening of rHuEPO. Left column shows the complete mutational screening of all positive charge amino acids (i.e. arginine and lysine) within the largest positively-charged patch (posQ) for aspartic acid (D). Next two columns summarize a set of substitutions of any amino acid in the posQ for D. The column on the right shows all the negative charge residues (i.e. aspartic and glutamic acid) within the posQ for arginine or lysine. Those proteins with posQ ratio above 1.0 are predicted as insoluble and below 1.0 as soluble. Selected proteins for further site-directed mutagenesis are highlighted in red. (PDF 168 kb) [file 12896_2019_520_MOESM3_ESM.pdf]

Additional file 3: Table S2. Summary of the solubility screening of rHuEPO. Left column shows the complete mutational screening of all positive charge amino acids (i.e. arginine and lysine) within the largest positively-charged patch (posQ) for aspartic acid (D). Next two columns summarize a set of substitutions of any amino acid in the posQ for D. The column on the right shows all the negative charge residues (i.e. aspartic and glutamic acid) within the posQ for arginine or lysine. Those proteins with posQ ratio above 1.0 are predicted as insoluble and below 1.0 as soluble. Selected proteins for further site-directed mutagenesis are highlighted in red.

Substitution of K-R in the posQ for D		Substitution of residues in the posQ for D				Substitution of D-E in the posQ for K-R	
**rHuEPO**	**posQ**	**rHuEPO**	**posQ**	**rHuEPO**	**posQ**	**rHuEPO**	**posQ**
**WT**	1.49	**WT**	1.49	**I133D**	1.40	**E13K**	2.471
**R4D**	1.29	**S9D**	0.89	**A135D**	1.39	**E13R**	2.222
**K20D**	1.23	**T27D**	1.39	**L141D**	1.40	**E23R**	1.63
**K45D**	0.68	**A30D**	1.42	**F142D**	1.39	**D136R**	1.71
**R139D**	1.28	**S34D**	1.42	**V144D**	1.38	**E159R**	1.79
**K140D**	1.26	**T44D**	1.35	**N147D**	1.19		
**R150D**	0.61	**N47D**	1.36	**F148D**	1.35		
**K152D**	0.75	**F48D**	0.75	**G151D**	0.74		
**K154D**	0.77	**Y49D**	1.43	**T157D**	0.87		
**R162D**	0.85	**W88D**	1.42	**G158D**	0.84		
